# Corrosion Characteristics of Polymer-Modified Oil Well Cement-Based Composite Materials in Geological Environment Containing Carbon Dioxide

**DOI:** 10.3390/polym16152187

**Published:** 2024-07-31

**Authors:** Yan Zhang, Junyu Xie, Weiming Zhao, Jie Dai, Fei Gao

**Affiliations:** 1 School of Petroleum Engineering, Yangtze University, Wuhan 430100, China; 2 State Key Laboratory of Petroleum Resources and Engineering, China University of Petroleum (Beijing), Beijing 102249, China; 3 Research Institute of Geology Xibu Drilling Engineering Company Ltd., CNPC, Kelamayi 834000, China; 4 CNOOC (China) Limited Tianjin Branch, Tianjin 300456, China

**Keywords:** oil well cement, corrosion, carbon dioxide, polymer material, response surface method

## Abstract

Oil well cement is easily damaged by carbon dioxide (CO_2_) corrosion, and the corrosion of oil well cement is affected by many factors in complex environments. The anti-corrosion performance of oil well cement can be improved by polymer materials. In order to explore the influence of different corrosion factors on the corrosion depth of polymer-modified oil well cement, the influence of different corrosion factors on corrosion depth was studied based on the Box–Behnken experimental design. The interaction of different influencing factors and the influence of multiple corrosion depths were analyzed based on the response surface method, and a response surface model was obtained for each factor and corrosion depth. The results indicate that within the scope of the study, the corrosion depth of polymer-modified oil well cement was most affected by time. The effects of temperature and the pressure of CO_2_ decreased sequentially. The response surface model had good significance, with a determination coefficient of 0.9907. The corrosion depth was most affected by the interaction between corrosion time and the pressure of CO_2_, while the corrosion depth was less affected by the interaction between corrosion temperature and corrosion time. Improving the CO_2_ intrusion resistance of cement slurry in an environment with a high concentration of CO_2_ gas can effectively ensure the long-term structural integrity of cement.

## 1. Introduction

Cement sheath is mainly used to seal off oil and gas reservoirs in developing oil and gas wells and geological storage wells of carbon dioxide [1,2,3]. The cement in oil wells is easily corroded by CO_2_ gas, causing damage to the microstructure of the cement sheath, and affecting the sealing integrity of the cement sheath. The cement sheath is prone to severe losses due to corrosion damage [4,5,6]. The downhole environment of oil well cement, with many influencing factors of corrosion, is complex. Different corrosion factors influence the corrosion of oil well cement in different ways, and the interaction between factors can also impact the corrosion of oil well cement. In order to improve the corrosion resistance of cement slurry, it is necessary to study the influence of different corrosion factors on cement slurry, and more scientific method guidance is needed when constructing the cement slurry system.

Polymer-modified cement-based composite materials for oil wells have been proven to have corrosion resistance. Elbakhshwan et al. [7] studied the modification mechanism of a novel self-healing polymer cement composite material in a CO_2_ environment, and found that the degree of carbonization of polymer cement is lower. Zhang, J. et al. [8] prepared environmentally responsive polymer microspheres to improve the corrosion resistance of cement paste. Zheng, Y. et al. [9] prepared a polymer called LJIY to solve the problem of cement ring cracking in acidic CO_2_ environments. The study found that LJIY has a significant effect on the healing of cement microcracks. Research has found that adding a certain amount of polymer materials can effectively improve the anti-corrosion ability of cement paste.

In their study of the corrosion of cement in acidic gas environments, J Liu et al. [10] studied the effect of carbonation on the microstructure of cement concrete and found that the depth of carbonation of cement increases with the water–cement ratio and carbonation age. B.L.S. Costa et al. [11] studied the corrosion changes in cement in an acidic gas environment and obtained a rule during the process of corrosion. T. Chen et al. [12] established a partial differential mathematical model for the coupling behavior of cement hydration and the carbonation reaction of cement pastes. K.C. Reddy et al. [13] used thermodynamic calculations to evaluate phase combinations of Portland cement with different clinker compositions, and concluded that calcite, zeolite, and gypsum are carbonated products. The CO_2_ content required for complete carbonation is directly related to the initial phase volume X. You et al. [14] introduced the carbonization process occurring in cement-based materials and its influencing factors and characterized the carbonization process of cement-based materials. H. Bao et al. [15] studied the degradation mechanism of cement-based materials in corrosive CO_2_ and H^+^ mixed solutions. The research shows that both the single and double corrosion of aggressive CO_2_ and H^+^ solutions significantly damage the quality of cement paste and concrete. The dual corrosion damage of corrosive CO_2_ and H^+^ to the test piece is more significant than that of a single corrosive CO_2_ solution or firm acidity. Y. Zhang et al. [16] studied the corrosion law of cement and found that the corrosion of cement in a CO_2_ environment is mainly affected by corrosion time, corrosion temperature, and CO_2_ concentration. The underground environment of oil and gas wells is complex and influenced by various factors. Currently, most of the research on oil well cement corrosion is based on indoor experiments and conducted by setting a single variable. There are few studies on the interaction between factors in oil well cement corrosion. The relationship between the corrosion factors of oil well cement and the impact of their interactions on the corrosion of oil well cement is temporarily unclear, and it is also difficult to observe the changes in oil well cement under long-term corrosion conditions. Therefore, studying and using scientific methods to analyze the impact of corrosion factors and corrosion changes in oil well cement is of great significance for comprehensively understanding the corrosion of oil well cement, predicting long-term corrosion changes in oil well cement, and designing corrosion-resistant cement slurry systems.

The response surface method is a statistical method that uses a scientific experimental design and multiple quadratic regression equations to fit the functional relationship between factors and response values [17]. S.K. Kaliyavaradhan et al. [18] obtained the optimal water–cement ratio using the response surface method to determine the CO_2_ absorption amount of WCP. S.P.S. Prabhakaran et al. [19] used response surface regression techniques to determine the effects of heating rate and temperature on mass loss, and studied the raw material characteristics of textile sludge in cement production. S.K. Nayak et al. [20] used a response surface model to conduct experimental and statistical evaluations of the relative impact of control factors on the erosion rate of composite materials, and evaluated the erosion and wear characteristics of waste marble-dust-filled polyester composites. Z.J. Zuo et al. [21] used analysis of variance (ANOVA) and response surface methodology to study the significance of individual factor effects and factor combinations, and concluded that the filling rate, impeller speed, and container speed showed an interaction with all presented responses. The response surface method can analyze the interaction of multiple factors by constructing a response surface of multiple factors and response values. Its constructed polynomial equation can also well reflect the fitting relationship between factors and response values, and can predict future changes. The response surface method is often used to study the optimization of materials and processes [22]. Researchers considered using the response surface to analyze the impact of corrosion factors on the corrosion of oil well cement based on the characteristics of the response surface method. The performance of oil well cement in complex acidic corrosion environments was analyzed based on the response analysis results. Currently, there needs to be more relevant research available in this field.

In order to study the changes in acidic corrosion of polymer-modified oil well cement in the formation environment of oil and gas wells, three main influencing factors of oil well cement corrosion were considered, and corrosion simulation experiments were conducted. The effect of a single factor on the corrosion depth of polymer-modified oil well cement was studied. Based on the Box–Behnken experimental design, response surfaces and quadratic polynomial prediction models were established for three corrosion factors and corrosion depths. The primary and secondary relationships between each experimental factor and response target, as well as the influence of the interaction between each factor on the degree of corrosion of polymer-modified oil well water sludge, were studied. Based on the analysis results, a method is proposed to improve the corrosion resistance of cement slurry. The research results can provide new methods for designing anti-corrosion cement slurries.

## 2. Materials and Methods

### 2.1. Materials

G-Grade oil well cement was used as the primary material for cement slurry produced by China Gezhouba Special Cement Plant, and its chemical composition is shown in Table 1. A filtrate reducer is mainly used to reduce the water loss of cement slurry, and it is a kind of 2-Acrylamido-2-methylpropane sulfonic acid polymer material. A retarder is used to adjust the thickening time, and it is a type of polyacid modified material. A dispersant can improve the fluidity of cement slurry, and it is an aldehyde ketone condensation polymer purchased from China Jingzhou Jiahua Technology Co., Ltd. (Jingzhou, China). An enhancer can be used to improve the strength of cement, and its main component is micro silicon. A polymer preservatives was used to improve the corrosion resistance, and its main component is waterborne resin emulsion. Its viscosity is 900cps and its solid content is 60%.

### 2.2. Methods

#### 2.2.1. Preparation and Testing of Cement Samples

The preparation of cement slurry were based on the corresponding methods. A constant speed mixer (TG-3060A, Shenyang Taige Petroleum Instrument and Equipment Co., Ltd., Shenyang, China) was used to mix evenly at 4000 rpm. The cement slurry composition is shown in Table 2.

The prepared cement slurry was placed in a cylindrical mold with a diameter of 25.4 mm and a height of 25.4 mm, and placed in a 60 °C water bath for constant temperature curing for 24 h to form an uncorroded solidified cement sample. The uncorroded cement sample was placed in a high-temperature and high-pressure corrosion tester for corrosion simulation experiments.

#### 2.2.2. Test of Corrosion Depth

**Figure 1:**
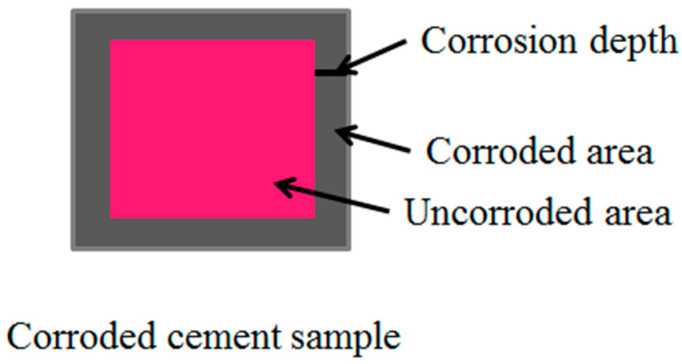
Cement sample corrosion diagram.

The alkaline Ca(OH)_2_ is the main hydration product of oil well cement [23]. The corrosion depth can be measured by calibrating the corrosion area using the characteristic that phenolphthalein reacts with alkaline substances in red color. A vernier caliper was used to measure the thickness values of the four edges of the sample that do not turn red, and the average value was taken as the corrosion depth of the cement sample, as shown in Figure 1.

#### 2.2.3. Single-Factor Corrosion Experiment Design

The cement corrosion tests were conducted based on three main influencing factors, with temperature, the pressure of CO_2_, and corrosion time as control variables. The corrosion temperature was controlled at five levels of 50 °C, 60 °C, 70 °C, 80 °C, and 90 °C, the pressure of CO_2_ was controlled at five levels of 5 MPa, 10 MPa, 15 MPa, 20 MPa, and 25 MPa, and corrosion time was controlled at five levels of 10 d, 20 d, 30 d, 40 d, and 50 d, respectively, to test the corrosion depth of cement samples after corrosion curing.

#### 2.2.4. Box Behnken Experimental Design

The Box–Behnken design has approximate rotatability, meaning that the predicted variance is only related to the distance from the experimental point to the design center, and is independent of its orientation. This means that in terms of prediction accuracy, the response variables of each point on the spherical surface with the design center as the center are the same. Three levels of the corrosion factors were selected, corrosion time (A): 30 d, 40 d, and 50 d; the pressure of CO_2_ (B): 15 MPa, 20 MPa, and 25 MPa; and corrosion temperature (C): 65 °C, 70 °C, and 75 °C. The corrosion depth (R1) of cement samples was selected as the response value.

Codes 1, 0, and −1 were set to explain the upper, middle, and lower levels of a single factor. The experimental factor design and level coding are shown in Table 3.

## 3. Result and Discussion

### 3.1. Basic Properties of Polymer-Modified Cement Slurry

**Figure 2:**
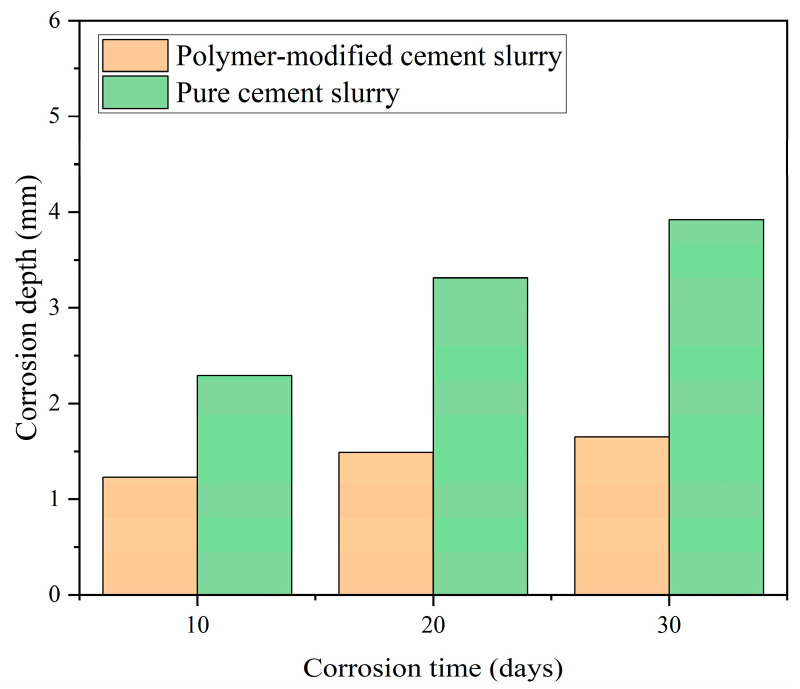
Corrosion depth of different samples at 20 MPa and 70 °C.

In order to clarify the basic performance of the designed polymer-modified anti-corrosion cement slurry, the experimental results of the performance evaluation of the cement slurry are shown in Table 4, and its microstructure is shown in Figure 1. From the experimental results, it can be seen that the studied polymer-modified cement slurry has a high flowability index and low consistency coefficient, which meets the requirements of fluidity for well cementing construction. At the same time, cement slurry has low water loss, high compressive strength, and suitable thickening time. Polymer-modified oil well cement has excellent basic performance, which is helpful for cementing construction. In addition, from the experimental results in Figure 2, it can be seen that the corrosion depth of polymer-modified cement slurry is significantly lower than that of pure cement slurry. When the corrosion time reached 30 days, the corrosion depth of polymer-modified cement slurry decreased by 57.9%. This is mainly because the addition of polymer resin can improve the compactness of cement samples, and, on the other hand, prevent hydration products from being corroded by carbon dioxide.

### 3.2. Analysis of Corrosion Factors in Polymer-Modified Cement Slurry

#### 3.2.1. Effect of Corrosion Time on Corrosion Depth of Polymer-Modified Oil Well Cement

**Figure 3:**
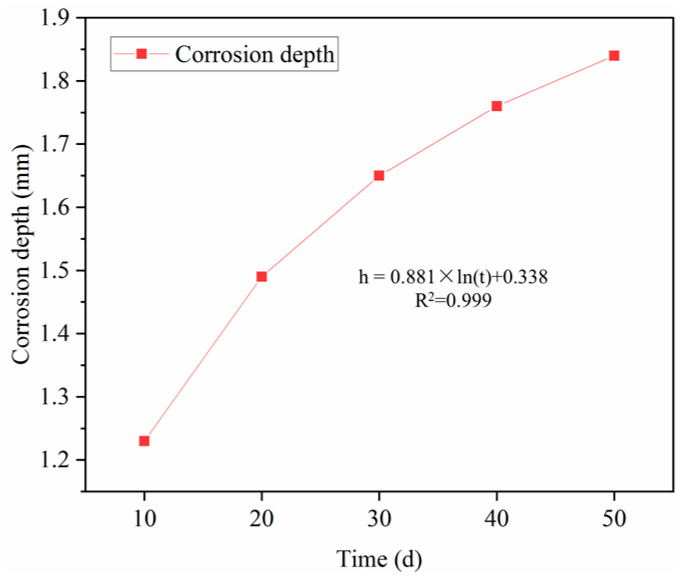
Effect of corrosion time on corrosion depth (20 MPa × 70 °C).

The effect of corrosion time on the corrosion depth of oil well cement was experimentally studied, and the experimental results are shown in Figure 3.

As seen from Figure 3, the corrosion depth of cement paste increases with the increase in corrosion time. When the corrosion time increased from 10 days to 30 days, the corrosion depth increased by 0.42 mm. After continuous corrosion for 50 days, the corrosion depth increased by 0.61 mm compared to 10 days of corrosion. The corrosion depth of cement and corrosion time roughly show a logarithmic growth relationship according to the curve change trend in corrosion depth and corrosion time. Alkaline Ca(OH)_2_ is continuously consumed during the corrosion process of oil well cement, and the resulting CaCO_3_ crystals loosen the structure of oil well cement, and increase porosity and permeability. The corrosion degree continues to increase as the corrosion time increases [24].

#### 3.2.2. Effect of Pressure of CO_2_ on Corrosion Depth of Polymer-Modified Oil Well Cement

**Figure 4:**
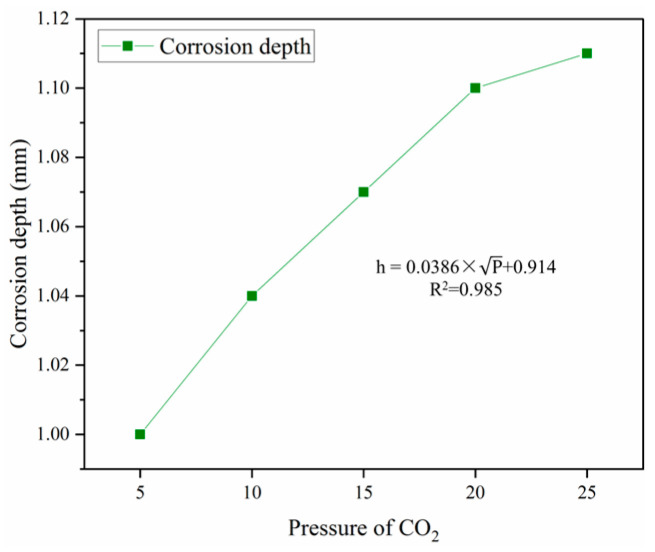
Effect of pressure of CO_2_ on corrosion depth (7 d × 70 °C).

The effect of the pressure of CO_2_ on the corrosion depth of oil well cement was experimentally studied, and the experimental results are shown in Figure 4.

As can be seen from Figure 4, the corrosion depth of cement stone increases with the increase in the pressure of CO_2_. The 7-day corrosion depth of cement stone increased by 0.11 mm, when the pressure of CO_2_ increased from 5 MPa to 25 MPa. The corrosion depth of cement and the pressure of CO_2_ assume a square root growth relationship according to the curve change trend in corrosion depth and the pressure of CO_2_. The content of CO_2_ will directly impact the corrosion of oil well cement during the corrosion process of oil well cement. The higher the CO_2_ content, the more significant the proportion of reaction with the hydration products of oil well cement in the environment, and the greater the degree of corrosion of oil well cement [25].

#### 3.2.3. Effect of Corrosion Temperature on Corrosion Depth of Polymer-Modified Oil Well Cement

**Figure 5:**
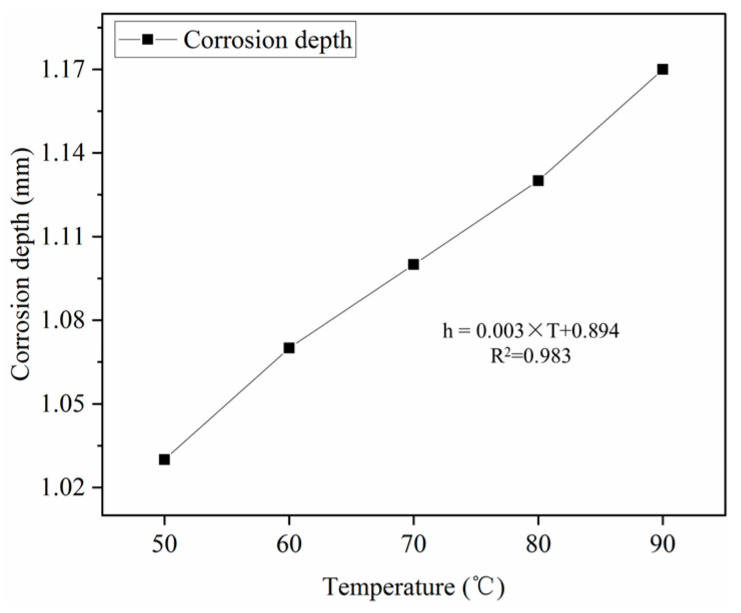
Effect of corrosion temperature on corrosion depth (20 MPa × 7 d).

The effect of corrosion temperature on the corrosion depth of oil well cement was experimentally studied, and the experimental results are shown in Figure 5.

As seen from Figure 5, the corrosion depth of cement increases with the increase in corrosion temperature. The 7-day corrosion depth of cement stone increased by 0.14 mm, when the corrosion temperature increased from 50 °C to 90 °C. The corrosion depth of cement paste and corrosion temperature show a roughly linear growth relationship according to the curve change trend in corrosion depth and temperature. Corrosion temperature will accelerate the reaction speed of corrosion during the corrosion process. The higher the temperature, the faster the reaction speed, and the greater the corrosion degree of oil well cement [26].

### 3.3. Corrosion Analysis of Polymer-Modified Cement Slurry under Multiple Factors

#### 3.3.1. Establishment of Response Surface Model

A binomial prediction model for the corrosion depth of cement paste and three factors is obtained by fitting experimental data in Table 5 [27].

R1 = 0.975833 + 0.014458 × A + 0.005917 × B + 0.002583 × C + 0.0002 × AB − 0.000025 × AC − 0.00015 × BC − 0.000092 × A^2^ + 0.000033 × B^2^ + 0.000033 × C^2^

The binomial equation considers the influence coefficient of each element, reflecting the influence of the interaction of single and dual factors on the corrosion depth of the cement sample.

F is a significance test indicator, and P is a probability, and they are used to jointly determine the significance of a regression model. The smaller P and larger F values reflect the significance and accuracy of the model. Mismatch terms reflect the significant degree of irrelevance between experimental data and the model. This indicates that the significant degree of model mismatch is higher with a P value less than 0.05 [28]. A statistically reliable regression model can be determined through analysis of variance.

#### 3.3.2. Analysis of Response Model

ANOVA was used to analyze the variance of the binomial equation and to test the significance of the error sources of the binomial model. The results of the analysis of variance are shown in Table 6.

In Table 6, the F value of the corrosion depth response surface regression model is 59.16, and the *p* value is <0.05. The model is significant at a 95% confidence level. The *p* value of the mismatched term 0.7370 is far greater than 0.05, and the mismatched term is not significant. The complex correlation coefficient R^2^ of the model is 0.9907, the corrected correlation coefficient R_adj_^2^ is 0.9740, and the coefficient of variation CV = 0.7147% < 10%, indicating that the selected model has high fitting accuracy and strong reliability [29]. The P values of the independent variables A, B, and C are all less than 0.05. The independent variables A, B, and C have a significant impact on the corresponding value R1, with the order of significance of the impact being corrosion time, pressure of CO_2_, and temperature, and the order of significance of the interaction between factors being corrosion time and pressure of CO_2_, pressure of CO_2_ and temperature, and corrosion time and temperature.

### 3.4. Interaction Effect Analysis of Various Factor

**Figure 6:**
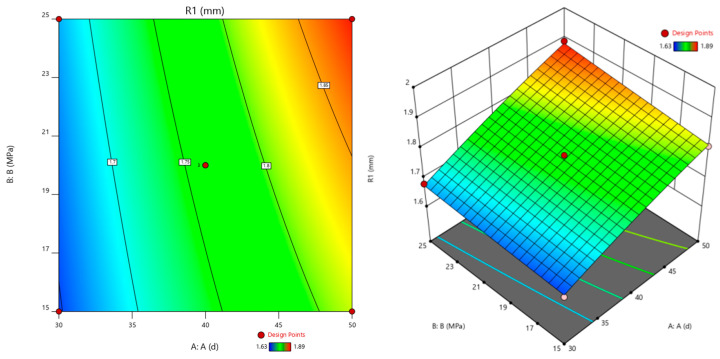
Response surface plots for the corrosion depth as a function of the corrosion time and the pressure of CO_2_ (corrosion temperature: 70 °C).

**Figure 7:**
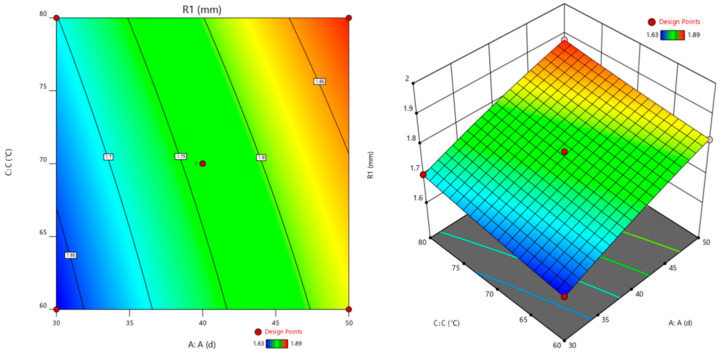
Response surface plots for the corrosion depth as a function of the corrosion time and the corrosion temperature (pressure of CO_2_: 20 MPa).

**Figure 8:**
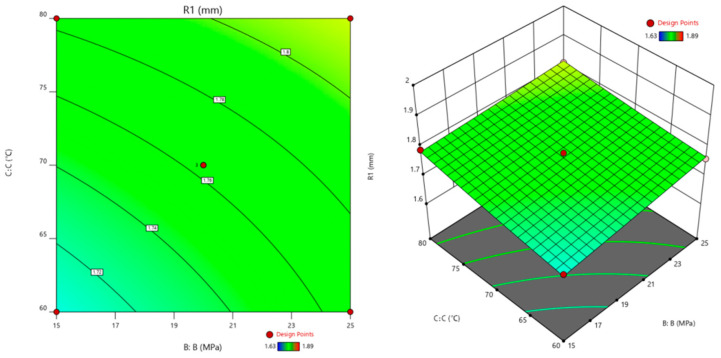
Response surface plots for the corrosion depth as a function of the pressure of CO_2_ and the corrosion temperature (corrosion time: 40 d).

The corrosion time (A), pressure of CO_2_ (B), and corrosion temperature (C) are used as the horizontal and vertical coordinates. The cement slurry corrosion depth (R1) is used as the *Z*-axis coordinate to construct a three-dimensional response surface graph, as shown in Figure 6, Figure 7 and Figure 8. The shape of the response surface and the contour line can illustrate the strength of the interaction between factors [30].

#### 3.4.1. Interaction Effect of the Corrosion Time and the Pressure of CO_2_

The interaction between corrosion time and the pressure of CO_2_ on the corrosion depth of cement is shown in Figure 6.

Figure 6 shows the interaction between corrosion time and the pressure of CO_2_ on the corrosion depth of cement. The curve of corrosion time is steeper, indicating that under experimental conditions, corrosion time has a more significant impact on the corrosion depth of cement paste, while the pressure of CO_2_ has a minor impact on the corrosion depth of cement. Under the condition of a high pressure of CO_2_, the corrosion depth of cement paste increases significantly with the increase in corrosion time. The interaction of corrosion time and CO_2_ pressure can accelerate the corrosion of oil well cement. The possible reason is that when the pressure of CO_2_ is high, as the corrosion time increases, CO_2_ with higher concentration can invade the oil well cement, causing more severe corrosion [31].

#### 3.4.2. Interaction Effect of Corrosion Time and Corrosion Temperature

The interaction between corrosion time and corrosion temperature on the corrosion depth of cement is shown in Figure 7.

Figure 7 shows the interaction between corrosion time and temperature on the corrosion depth of cement. The steep curve of corrosion time indicates that the formula used in the experiment has a significant impact on the corrosion depth of cement paste under experimental conditions, while the impact of corrosion temperature on the corrosion depth of cement paste is relatively tiny. At higher corrosion temperatures, with the increase in corrosion time, the corrosion depth of cement paste slowly increases. The interaction of corrosion time and temperature makes the early corrosion of oil well cement faster, and with the increase in corrosion time, the corrosion depth changes relatively slowly. The possible reason is that the corrosion rate of oil well cement under acidic conditions is relatively fast when the temperature is high. The CaCO_3_ crystal generated during corrosion expands, making the structure of the cement sample relatively dense, making it difficult for corrosive media to invade, and the corrosion rate is relatively slow [23].

#### 3.4.3. Interaction Effect of the Pressure of CO_2_ and Corrosion Temperature

The interaction between the pressure of CO_2_ and corrosion temperature on the corrosion depth of cement is shown in Figure 8.

Figure 8 shows the interaction between the pressure of CO_2_ and corrosion temperature on the corrosion depth of cement. The corrosion temperature curve is steeper, indicating that the formula used in the experiment significantly impacts the corrosion depth of cement paste under experimental conditions. At higher corrosion temperatures, as the pressure of CO_2_ increases, the corrosion depth of cement changes relatively gently. The CaCO_3_ crystal formed by the rapid reaction of oil well cement at higher temperatures makes its structure relatively dense. The CO_2_ content increases, but the content involved in the corrosion reaction is relatively small, and the corrosion depth changes slowly. When the temperature is low, the corrosion rate of oil well cement is relatively high as the CO_2_ content increases. When the CO_2_ content is low, the oil well cement undergoes corrosion changes, with some hydration products corroded. As the CO_2_ concentration increases, more CO_2_ participates in the reaction, and the corrosion rate of oil well cement gradually increases [32].

### 3.5. Analysis of Anti-Corrosion Effect of Polymers on Cement

#### 3.5.1. Corrosion of Oil Well Cement

Oil well cement belongs to a type of Portland cement, and its main components are tricalcium silicate C_3_S (3CaO·SiO_2_), dicalcium silicate C_2_S (2CaO·SiO_2_), tricalcium aluminate C_3_A (3CaO·Al_2_O_3_), and tetracalcium ferroaluminate C_4_AF (4CaO·Al_2_O_3_·Fe_2_O_3_) [33]. Cement mainly generates calcium hydroxide, hydrated calcium silicate, and silicate minerals during the hydration process. The hydration reaction is as follows:

C_3_S + 4H_2_O → 10/9C_1.5_S_0.9_H_2.4_ + 4/3CH (1)

C_2_S + 3H_2_O → 10/9C_1.5_S_0.9_H_2.4_ + 1/3CH (2)

C_3_A + 6H_2_O → C_3_AH_6_ (3)

C_4_AF + 10H_2_O → C_3_AH_6_ + CH + FH_3_ (4)

The various hydration products formed during the cement hydration process pile up with each other, forming a dense cement stone structure, which protects the casing from damage caused by formation stress. When there is acidic CO_2_ gas in the formation, the acidic gas dissolves in the formation water, forming an acidic liquid environment. There is a large amount of calcium hydroxide in the hydration products of oil well cement. In the acidic environment, cement undergoes corrosion and damage, leading to increased porosity and loose structure. In severe cases, it may also cause damage. The corrosion and damage process are as follows [34]:

CO_2_ + H_2_O → H_2_CO_3_ → HCO_3_^−^ + H^+^ (5)

Ca(OH)_2_ + H^+^+HCO_3_^−^ → CaCO_3_ + 2H_2_O (6)

CSH + H^+^ + HCO_3_^−^ → CaCO_3_ + SiO_2_ + H_2_O (7)

CO_2_ + H_2_O + CaCO_3_ → Ca(HCO_3_)_2_ (8)

As time goes on, the formation water transports calcium ions away during the flow process, causing continuous corrosion and damage to the cement stone. The analysis results show that as the temperature increases, the corrosion reaction rate in the oil well cement accelerates, and its corrosion depth relatively increases. With the increase in carbon dioxide pressure, more acidic corrosive media are more likely to invade the cement, damaging the structure of the cement stone. As time goes on, more calcium hydroxide is lost inside the cement stone, causing more significant corrosion damage to the cement stone. Under the interaction of multiple factors, the corrosion damage inside the cement stone is more severe.

#### 3.5.2. Mechanism of Polymer-Reinforced Anti-Corrosion Property of Oil Well Cement

**Figure 9:**
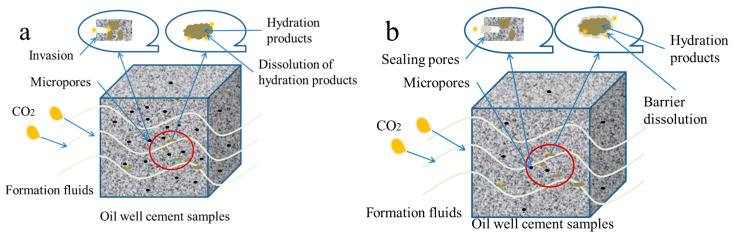
The corrosion of oil well cement (**a**) and the way polymer enhances the anti-corrosion performance of oil well cement (**b**).

Oil well cement is generally used in deeper formations, and the method of applying anti-corrosion coatings on buildings is not suitable for the anti-corrosion of oil well cement. Research has found that polymer additives can effectively improve the anti-corrosion performance of oil well cement. After adding a certain amount of polymer material, cement stone can form a denser structure with lower porosity and can resist the invasion of acidic corrosive gasses. At the same time, polymer additives can form polymer films to encapsulate hydration products in oil well cement, further slowing down the corrosion of cement hydration products [35], as shown in Figure 9.

Through the analysis of the corrosion changes in cement samples under the interaction of multi factors in Figure 6, Figure 7 and Figure 8 and the mechanism analysis of enhanced anti-corrosion performance of cement samples by polymer materials in Figure 9, it can be concluded that the corrosion rate of cement stone is relatively accelerated under higher temperature and pressure. The corrosion depth significantly increases under the interaction of corrosion pressure and corrosion time. In order to improve the anti-corrosion ability of cement stone, polymer anti-corrosion agents should be added in the design of cement slurry system, which can prevent the invasion of acidic corrosive media and improve the dense structure inside the cement stone to enhance its corrosion resistance.

## 4. Conclusions

(1) Polymer-modified oil well cement has good basic performance, which meets the requirements of cementing operations. Meanwhile, its ability to resist corrosion is significantly better than that of blank cement slurry.
(2) Polymer-modified oil well cement is significantly affected by corrosion time, the pressure of CO_2_, and corrosion temperature. According to the fitting data results, the corrosion depth is logarithmic with the corrosion temperature, square root with the pressure of CO_2_, and linear with the corrosion time.
(3) Under experimental conditions, it can be seen that polymer-modified oil well cement is most affected by the interaction between the pressure of CO_2_ and corrosion time under the influence of various corrosion factors in complex formations. The long-term corrosion resistance of oil well cement can be enhanced by improving the structural density of cement stone and blocking the invasion of acidic corrosive media by adding polymer preservatives.
(4) The corrosion changes in polymer-modified oil well cement under the interaction of multiple complex factors can be well explained using the response surface model of corrosion depth based on multiple corrosion factors, and the impact of the interaction between factors on the corrosion of oil well cement can be facilitated and understood, providing guidance for the long-term anti-corrosion design of oil well cement.

## Figures and Tables

**Table 1:** Chemical components of oil well cement.

Component	CaO	SiO_2_	Al_2_O_3_	Fe_2_O_3_	MgO	SO_3_	Other
Content (wt%)	64.11	22.40	3.62	4.42	1.24	2.23	1.98

**Table 2:** Components of G-class oil well cement.

Samples	Content (wt%)						
	Cement	Water	Enhancer	Dispersant	Filtrate Reducer	Retarder	Polymer Preservative
Polymer-modified cement slurry	100	40	6	0.6	4	0.5	12
Pure cement slurry	100	40	6	0.6	4	0.5	0

**Table 3:** Variable level design and coding.

Real Variables	Code	Code Level		
		−1	0	1
Time (d)	A	30	40	50
Pressure of CO_2_ (MPa)	B	15	20	25
Temperature (°C)	C	60	70	80

**Table 4:** Basic properties of polymer-modified cement slurry tested at 70 °C.

Performance	Test Results
Flowability index	0.86
Consistency coefficient (Pa·S^n^)	0.27
Compressive strength (MPa)	24.3
Water loss (mL)	38
Thickening time (min)	217

**Table 5:** Box–Behnken test scheme and results.

No.	Code Value			Corrosion Depth (mm)		Error	
	A	B	C	Actual Value	Predicted Value	Absolute Error (mm)	Relative Error (%)
1	40	20	70	1.75	1.763	0.013	0.74
2	40	15	60	1.71	1.704	−0.006	−0.35
3	40	25	60	1.76	1.766	0.006	0.34
4	30	20	60	1.63	1.629	−0.001	−0.06
5	40	20	70	1.78	1.763	−0.017	−0.96
6	30	25	70	1.68	1.675	−0.005	−0.30
7	30	20	80	1.7	1.699	−0.001	−0.06
8	50	15	70	1.81	1.815	0.005	0.28
9	50	20	60	1.82	1.821	0.001	0.05
10	50	25	70	1.89	1.883	−0.007	−0.37
11	40	20	70	1.76	1.763	0.003	0.17
12	30	15	70	1.64	1.648	0.008	0.49
13	40	25	80	1.81	1.816	0.006	0.33
14	50	20	80	1.88	1.881	0.001	0.05
15	40	15	80	1.79	1.784	−0.006	−0.34

Notes: Relative error= (Absolute error/Actual value) × 100%; Absolute error = Actual value − Predicted value.

**Table 6:** Variance analysis of regression model for corrosion depth.

Source	Sum of Squares	Df	Mean Square	F-Value	*p*-Value	Significance
Model	0.0843	9	0.0094	59.16	0.0002	Significant
A	0.0703	1	0.0703	444.08	<0.0001	
B	0.0045	1	0.0045	28.50	0.0031	
C	0.0084	1	0.0084	53.37	0.0008	
AB	0.0004	1	0.0004	2.53	0.1728	
AC	0.0000	1	0.0000	0.1579	0.7075	
BC	0.0002	1	0.0002	1.42	0.2867	
A²	0.0003	1	0.0003	1.96	0.2205	
B²	0.0000	1	0.0000	0.0162	0.9037	
C²	0.0000	1	0.0000	0.2591	0.6324	
Residual	0.0008	5	0.0002			
Lack of Fit	0.0003	3	0.0001	0.4643	0.7370	Not significant
Pure Error	0.0005	2	0.0002			
Cor Total	0.0851	14				

## Data Availability

Data will be available upon request.
